# Anatomic Brain Asymmetry in Vervet Monkeys

**DOI:** 10.1371/journal.pone.0028243

**Published:** 2011-12-21

**Authors:** Scott C. Fears, Kevin Scheibel, Zvart Abaryan, Chris Lee, Susan K. Service, Matthew J. Jorgensen, Lynn A. Fairbanks, Rita M. Cantor, Nelson B. Freimer, Roger P. Woods

**Affiliations:** 1 Department of Psychiatry and Biobehavioral Sciences, The Semel Institute for Neuroscience and Human Behavior, University of California, Los Angeles, Los Angeles, California, United States of America; 2 Center for Neurobehavioral Genetics, The Semel Institute for Neuroscience and Human Behavior, University of California, Los Angeles, California, United States of America; 3 David Geffen School of Medicine, University of California, Los Angeles, Los Angeles, California, United States of America; 4 Ahmanson-Lovelace Brain Mapping Center, University of California, Los Angeles, Los Angeles, California, United States of America; 5 Department of Pathology/Comparative Medicine, Wake Forest University Health Sciences, Winston-Salem, North Carolina, United States of America; 6 Department of Human Genetics, University of California, Los Angeles, Los Angeles, California, United States of America; 7 Department of Neurology, University of California, Los Angeles, Los Angeles, California, United States of America; Nathan Kline Institute and New York University School of Medicine, United States of America

## Abstract

Asymmetry is a prominent feature of human brains with important functional consequences. Many asymmetric traits show population bias, but little is known about the genetic and environmental sources contributing to inter-individual variance. Anatomic asymmetry has been observed in Old World monkeys, but the evidence for the direction and extent of asymmetry is equivocal and only one study has estimated the genetic contributions to inter-individual variance. In this study we characterize a range of qualitative and quantitative asymmetry measures in structural brain MRIs acquired from an extended pedigree of Old World vervet monkeys (n = 357), and implement variance component methods to estimate the proportion of trait variance attributable to genetic and environmental sources. Four of six asymmetry measures show pedigree-level bias and one of the traits has a significant heritability estimate of about 30%. We also found that environmental variables more significantly influence the width of the right compared to the left prefrontal lobe.

## Introduction

The high degree of lateralization in the capacity for some behaviors has stimulated decades of investigation regarding anatomic brain asymmetry, much of which has focused on the evolution of regions hypothesized to relate to language. Comparative studies in primates have shown that the human tendency for leftward biased asymmetry of Broca's area and the planum temporale is present in chimpanzees and gorillas [1]–[5], but is not present in Old World monkeys [6]–[9]. Outside of these specific regions, the evidence for anatomical asymmetry in monkeys is equivocal. Although several studies have not found asymmetry [6], [9], [10], other studies have identified asymmetric features in several species of Old World monkeys [11]–[14]. The interpretation of the findings is difficult because the direction of apparent asymmetry differs between studies. Some studies have found rightward asymmetry in the frontal lobes [13], [15], whereas other studies suggest leftward asymmetry in the frontal lobes [12], [14]. In this study, we address some of the limitations of previous studies and identify pedigree-level anatomic asymmetry in another species of Old World monkey, *C. aethiops* (commonly termed African green monkeys or vervets).

Several factors have contributed to the equivocal findings in previous studies of anatomic asymmetry in monkeys. With the notable exception of a large sample from the Cayo Santiago macaque colony [13], [15], most study designs have included a relatively limited representation of individuals from a particular species. Another factor that has confused the comparative brain asymmetry literature is the lack of standardization in how asymmetry phenotypes are defined. Observations have been collected from post-mortem brains [9], [10], [14], endocranial casts [13], [15], photographs [12], [14] and MRIs [8], [16], [17] and asymmetry measures have been derived from lobar volume [8], sulcal morphology [9], [10], [12]–[14], [17], lobar protrusions (petalias) [13], [15] and cerebral widths [6]. In the current study we examined six measures of anatomic asymmetry in a large sample (n = 357) of high resolution structural MRIs collected from all adult members of the UCLA/Wake Forest Vervet Research Colony (VRC). We selected one qualitative feature (relative position of the cingulate sulcus) and five quantitative measures of asymmetry to provide broad coverage for detecting anatomic asymmetry and we show that four of the six measures are asymmetric at the pedigree level.

An additional focus of asymmetry research has been the attempt to identify the underlying basis of inter-individual variation in anatomic asymmetry. Several studies in humans have used twin designs to show that genes and environment differentially affect the volumes of the left and right hemispheres [18], [19]. These studies did not measure asymmetry *per se*, but showed a lateralized influence of genes and environment on the two hemispheres. To our knowledge no studies in humans have examined genetic and environmental contributions to global measures of anatomic asymmetry and only one study has been performed in NHPs, which used indirect measurements of brain asymmetry from post-mortem skulls in macaques and found a modest genetic contribution (∼30%) to prefrontal asymmetry [15]. In the current study, we leverage the power of a complex extended pedigree to estimate the genetic and environmental contributions to anatomic asymmetry and show a heritability of around 30% for one of the asymmetry phenotypes.

## Methods

### 1 Subjects, MRI acquisition and Image Processing

#### 1.1 Ethics Statement

In accordance with the recommendations of the Weatherall report, “The use of non-human primates in research” the following details are provided regarding animal welfare and steps taken to ameliorate suffering of the vervet monkeys. Studies were conducted as approved by the Animal Research Committee in the Office for Protection of Research Subjects at UCLA and the Institutional Animal Care and Use Committee at the Sepulveda Veterans Administration Medical Center. The vervet subjects scanned in this study are part of an extended pedigree of the UCLA/Wake Forest Vervet Research Colony (VRC) which has included more than 1000 individuals since its founding in the 1970's. The animals were housed in large outdoor/indoor enclosures, each with between nine and 41 animals in groups designed to model natural vervet social organization. Food and water is readily available and disruptions to the colony are minimized to the extent possible. The brain images use in this study were acquired non-invasively using MRI technology. The only invasive procedure performed was the establishment of a venous catheter for the administration of anesthetics during image acquisition. Catheter placement was performed under aseptic conditions, and animals were monitored continuously during anesthesia to assure maintenance of normal physiological parameters. Animals were fasted starting the night prior to the procedure to reduce risk of aspiration. After initial intramuscular administration of 15 mg/kg ketamine, an intravenous line was established and used to initiate anesthesia using midazolam (0.25 mg/kg) and ketamine (5 mg/kg). Each animal was also given 0.027 mg/kg atropine and intubated to protect the airway and reduce risk of aspiration. After intubation, respiratory rate was monitored frequently and used to maintain adequate anesthesia using midazolam and ketamine. After the MRI procedure, anesthesia was discontinued and all animals were directly observed until they recovered completely from the anesthesia and returned to normal functioning. All animals tolerated the procedure well and there were no adverse events associated with any of the experiments.

#### 1.2 MRI acquisition and Image Processing

Details of the image acquisition and pre-processing protocol have been described previously [20]. Briefly, nine separate structural scans were acquired from 357 animals (256 females and 101 males) using an 8-channel high-resolution knee array coil as a receiver in a 1.5 Tesla Siemens (Erlanger) Symphony unit. The images were acquired as axial T1-weighted volumes with a 3D magnetization prepared rapid acquisition gradient echo (MPRAGE). The TR was 1900 msec, the TE 4.38 msec and the TI 1100 msec. A flip angle of 15 degrees was used. Voxel resolution was 0.5 mm in all three planes. The nine separate images were aligned to each other in pair-wise rigid body registrations and averaged together to yield one high signal-to-noise image.

After removal of image regions corresponding to non-brain tissue, an affine population atlas was created from the individual MRI images using methods described by Woods [21]. A final symmetric template was generated by reflecting the population atlas across the x-axis (left-right mirroring) and averaging it with the non-reflected image to generate an atlas with absolute right-left symmetry. To assure that no subtle right-left biases were introduced by the algorithm used to register images, all images were reregistered to this final symmetric atlas using an in-house version of the AIR5.2.5 alignlinear registration algorithm that guarantees left-right invariance by reflecting the pair of images being registered across the eight possible combinations of axis orientations (x, y, z, xy, xz, yz, and xyz) during the minimization procedure of the registration algorithm. The native space segmented brain images were then resliced directly into this final ‘mirror invariant’ affine space and used for subsequent volumetric analysis. To examine the relative position of the cingulate sulcus and to determine cerebral widths, rigid body transformations, derived from the affine space registrations using singular value decomposition, were used to resample images into a rigid body common space prior to phenotyping.

### 2 Qualitative Measure: Ascending Ramus of the Cingulate Sulcus

**Figure 1:**
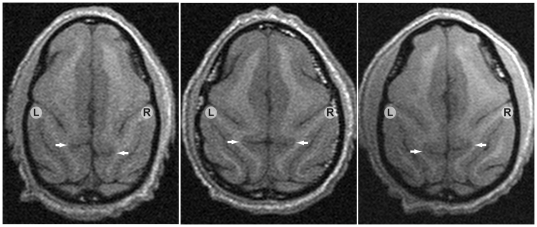
Cingulate sulcus asymmetry. Transverse Slices showing the relative position of the left and right ascending ramus of the cingulate sulcus. The left panel shows an image that was consistently scored as +2, the middle panel shows an image scored as symmetric (score = 0), and the right panel shows an image scored as −2.

**Figure 2:**
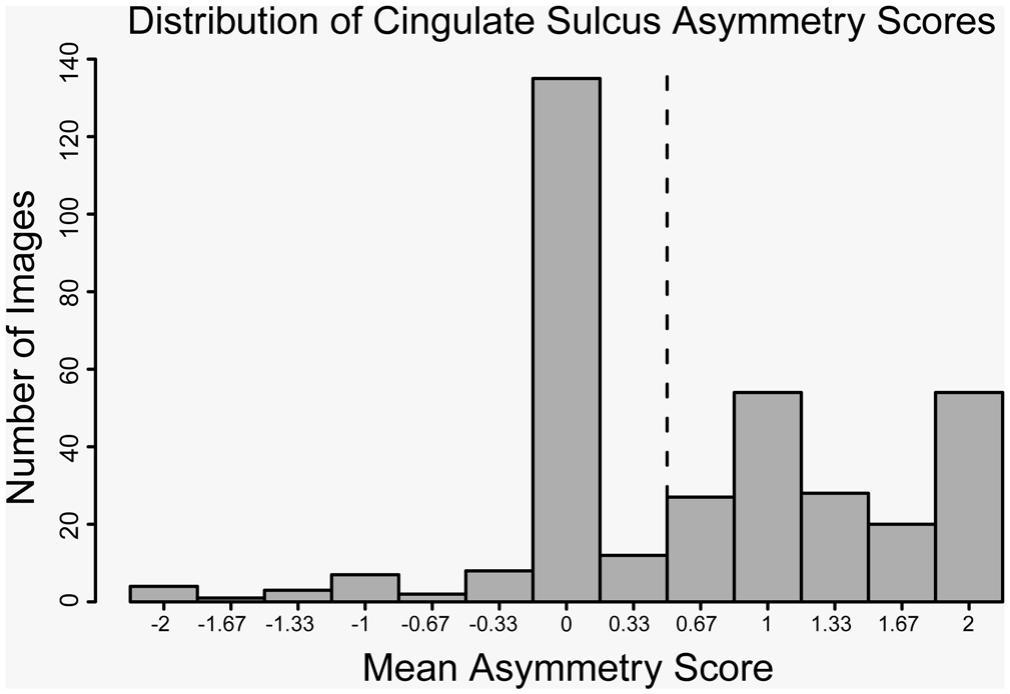
Distribution of cingulate sulcus asymmetry scores, averaged across three ratings. The dotted line indicates the threshold for defining symmetric (left of line) and asymmetric (right of line) phenotypes.

A single transverse slice dorsal to the upward deflection of the cingulate sulcus was selected from each image that showed the relative positions of the ascending rami of the left and right cingulate sulcus at the midline of the cerebrum. The left-right orientation of each slice was randomized by reflecting half of the images across the x-axis (left-right mirroring). Each slice was then scored three times by an expert rater (R.P.W) on a scale between −2 and +2 based on the relative positions of the cingulate sulci at the midline; 0 = symmetric (Figure 1, middle panel), −2 = strong asymmetry with right sulcus anterior to the left (Figure 1, right panel), +2 = strong asymmetry with left sulcus anterior to the right (Figure 1, left panel). Scores for the same image showed high correspondence between the three ratings (see distribution of scores, Figure 2). The inter-rater reliability of these scores was confirmed on a subset of the images by three independent, blinded raters.

### 3 Quantitative Measures

#### 3.1 Hemisphere Volume Analysis

The registration procedure used to transform each brain into a common space ensured that the mid-sagittal plane of each image was located in the mid-line of the image matrix and allowed precise separation of the left and right hemispheres. This fact was empirically checked by repeating the image registration protocol using right-left reflected images to ensure that the hemispheric volumes were exactly reversed as expected. Hemispheric volumes determined in affine space were converted back to native space dimensions prior to analysis. An asymmetry quotient (AQ) was calculated by subtracting the value of the left side from the value of the right side and dividing the result by the average value of the two hemispheres:
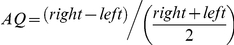
An AQ near zero indicates symmetry, whereas a positive AQ indicates asymmetry with a larger right hemisphere and a negative AQ indicates asymmetry with a larger left hemisphere.

#### 3.2 Cerebral Width

**Figure 3:**
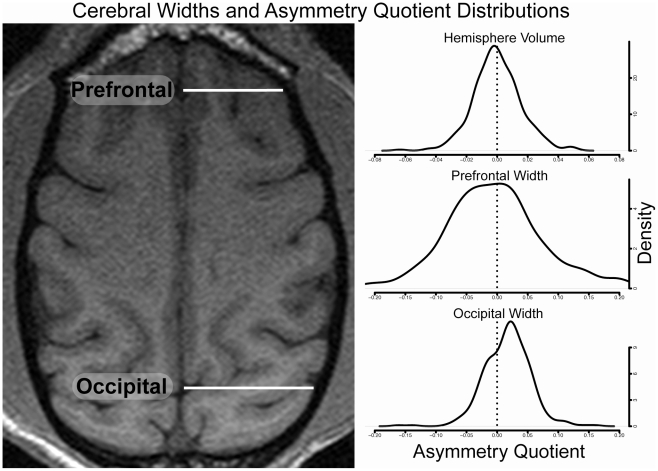
Cerebral Widths and Asymmetry Quotient Distributions. Left panel shows an example of a traverse slice dorsal to the corpus callosum with cerebral widths indicated on the right hemisphere. Right panel shows the distributions of the asymmetry quotients for hemisphere volume and each cerebral width.

After rigid-body transformation into a common space, a single transverse slice directly dorsal to the corpus callosum was selected from each image. This slice was chosen to ensure the inter-hemispheric separation was distinct across the anterior-posterior axis. Half of the slices were randomized with respect to left-right orientation by mirroring them across the mid-sagittal plane. All slices were reassembled randomly into a single 3D volume for phenotyping, which was performed using OsiriX imaging software version 3.7.1 (http://www.osirix-viewer.com/). Cerebral width was measured for each hemisphere at two locations (Figure 3). Cerebral width for the occipital hemispheres was defined from the parieto-occipital sulcus at the midline to the lateral edge of the cerebrum. The width of the prefrontal region was measured from the anterior cingulate sulcus at the midline to the lateral edge of the frontal lobe. Every cerebral width was assessed independently by two researchers (D.C. and J.C.), whose measurements were highly correlated (r = 0.99) and averaged to yield the final phenotype. The asymmetry quotients for the occipital and prefrontal widths were calculated as described above.

#### 3.3 Atlas-Based Phenotype: Skew

**Figure 4:**
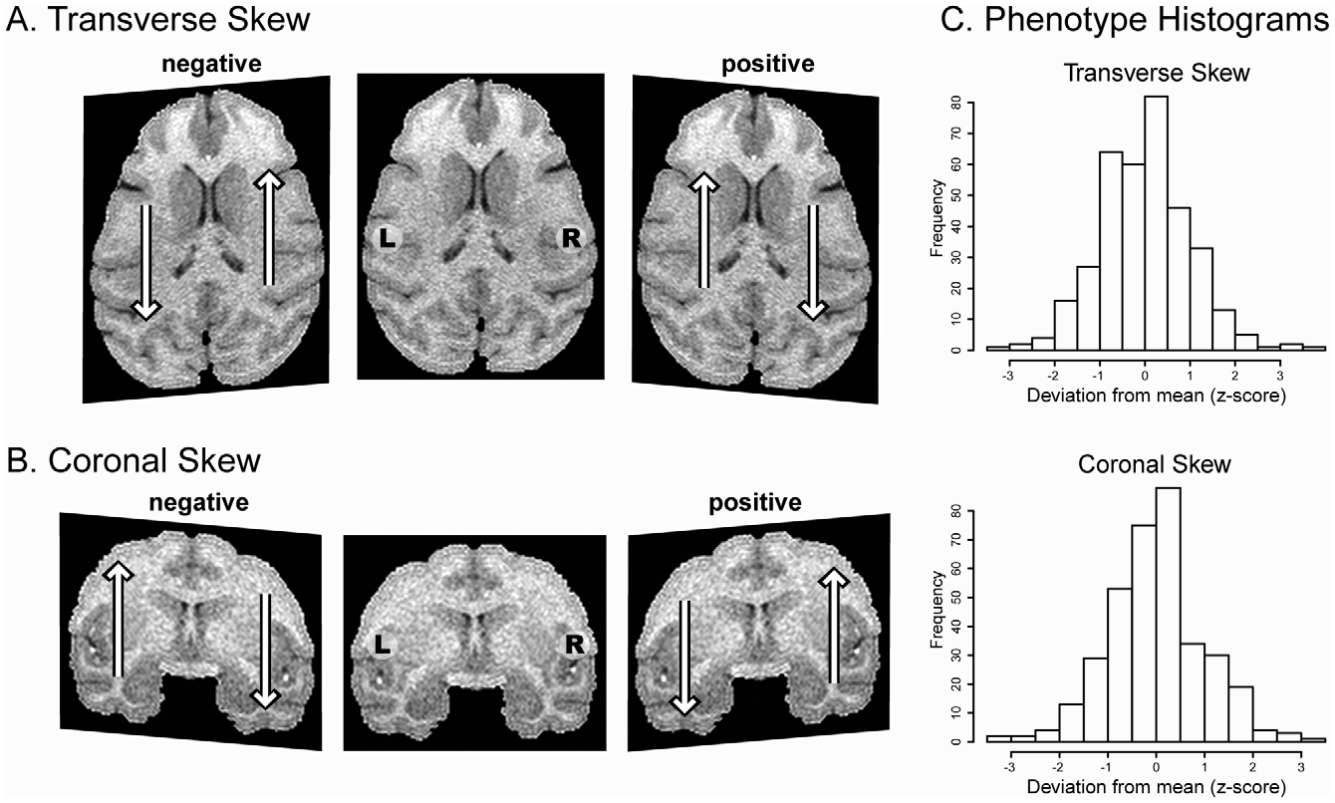
Deformation phenotypes. During the affine registration process, native space images are skewed (sheared) to ‘correct’ hemispheric asymmetry and align the images to the symmetric template. The magnitude of the skewing is a quantitative measure of hemispheric asymmetry. The arrows in each panel indicate the direction volume is shifted during image registration. The asymmetric distribution of volume in the native space (non-deformed) image is therefore opposite to the direction of the arrows. The skews have been exaggerated to emphasize the otherwise subtle distortions introduced by the registration process. Panel A: A positive skew in the transverse plane corresponds to an anterior shift of voxels in the left hemisphere and a posterior shift of voxels in the right hemisphere during registration to the symmetric template. Panel B: A positive skew in the coronal plane leads to a ventral shift of voxels in the left hemisphere and a dorsal shift of voxels in the right hemisphere during registration to the symmetric template. Panel C shows the distributions of the normalized phenotypes.

The affine transformations generated by the registration process capture information about global morphometric differences between the vervet brain images. The magnitude of skewing for each plane was determined from the linear transformations mapping each image to the symmetric template using programs available in the AIR 5.2.5 software package [21]. Of particular interest for characterizing asymmetry is the skewing (shearing) in the coronal and transverse planes (Figure 4, Panels A and B) that is required to register an individual image to the right-left symmetric template. During image registration, asymmetry in the native space image is ‘corrected’ when the image is deformed to match the symmetric template. The arrows in each panel of Figure 4 show the direction volume is shifted in each hemisphere during the registration process indicating that the direction of asymmetry in the native space image is opposite to the affine deformation. Therefore a positive skew in the transverse plane is produced by a brain with volume asymmetrically distributed toward the left in posterior (occipital) regions and asymmetrically shifted to the right in anterior (prefrontal) regions (Figure 4, Panels A). A positive skew in the coronal plane suggests that the brain has a global morphometry shifted towards the dorsal left hemisphere and shifted towards the ventral right hemisphere (Figure 4, Panels B).

### 4 Statistical Analysis

The extensive inter-relatedness of individuals in this study violated the independence assumptions for standard statistical analyses. Consequently, permutation methods were used to generate null distributions for each asymmetry phenotype and then test whether the measure was significantly different from that expected under the null-hypothesis. Null distributions for the volume and width AQ phenotypes were generated by randomly shuffling measurements from the left and right side for each individual before calculating an AQ and averaging for the entire shuffled sample. For the skew phenotype, each image was reflected across the left-right axis and registered to the symmetric template before determining the skewing phenotype as described above. The null distributions were estimated by randomly selecting a native-space or mirrored-space skew phenotype from each individual before determining the sample average. The permutation process was repeated 100,000 times for each phenotype. A ‘two-tailed’ approach was used to count the number of permutations that produced an estimated statistic extreme to the sample estimate (i.e. greater than the sample estimate or less than the negative of the sample estimate). This count was divided by 100,000 to determine the p-value. A similar approach was used to determine the significance of the correlation coefficient estimates between phenotypes. The null distribution for each correlation estimate was generated by randomly shuffling each pair of phenotype vectors before estimating the coefficient. This process was repeated 100,000 times and p-values were calculated as above.

### 5 Variance Component Analysis of Genetic and Environmental Influences

Additive genetic heritability (*_h_* ^2^) represents the proportion of total phenotypic variance (σ^2^_Z_) attributable to all genetic sources (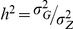), where σ^2^_G_ represents the genetic variance. Total phenotypic variance is constrained to 1.0, therefore environmental variance (σ_E_) can be determined from the heritability estimate (σ_E_ = 1−σ_G_) and represents the proportion of variance that is due to all non-genetic sources. The large size of the VRC pedigree provided good power to obtain significant estimates of the genetic contribution to anatomic asymmetry. The SOLAR package [22] uses a variance components approach to estimate the additive polygenic component of variation considering the entire pedigree jointly. The pedigree structure is used to build a matrix of kinship coefficients (Φ*_ij_*) for each pair of individuals in the pedigree, where each entry represents the probability that two genes, sampled at a particular locus from each individual are identical and inherited from a common ancestor. A maximum likelihood approach is implemented to estimate σ^2^_G_ from the phenotypic covariance: 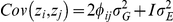 where *z_i_* represents the phenotypic value for the i^th^ individual and *I* is the identity matrix. SOLAR also implements bivariate polygenic analyses to estimate genetic (ρ_G_) and environmental correlations (ρ_E_) from total phenotypic correlation (ρ_P_). Genetic correlation is an estimate of the proportion of the covariance of two phenotypes that is due to common genetic sources and is a direct measure of the pleiotropic contributions to two traits (i.e. genetic loci that influence both traits). Likewise, the estimate of environmental correlation represents the common sources of non-genetic causal influences contributing to the covariance between two phenotypes. The two correlations are estimated using the following model: 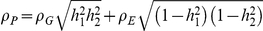, where *h^2^_1_* and *h^2^_2_* are the heritabilities of the two traits. Prior to genetic analysis, standard linear regression was used to model the effects of sex, age and their interaction using the R environment [23]. Heritability analysis was performed on the residuals from the linear models. The inverse normal function as implemented in SOLAR was used to transform quantitative variables that did not satisfy the Shapiro-Wilk test for normality.

## Results

We acquired six asymmetry phenotypes from each brain image. We examined one sulcal feature, the relative position of the left and right ascending rami of the cingulate sulcus, which was chosen because the ascending rami lie approximately between the frontal and parietal lobes and are therefore influenced by the anterior-posterior distribution of cerebral volume. Furthermore, the relative positions of the ascending rami are readily and reliably scored on transverse slices and we observed biased asymmetry for this trait in thirteen of twenty-two pilot animals scanned separately prior to the larger study. In addition to the qualitative sulcal feature, we generated quantitative phenotypes for hemispheric volume and prefrontal and occipital width. We also implemented a novel measure of asymmetry using probabilistic atlas-based methods to assess the magnitude of skew in the transverse and coronal planes required to deform each image to align it to a symmetric atlas-template. This atlas-based method uses information from all voxels in the image and provides a global measure of anatomic asymmetry. Below, we first present the analysis of each individual phenotype followed by an analysis of the correlations between the different measures. Finally we estimate the proportion of variance for each phenotype that is attributable to genetic and environmental causes.

### 1 Phenotypes

#### 1.1 Ascending Ramus of Cingulate Sulcus

Each brain image was scored three times for relative position of the ascending ramus of the cingulate sulcus in each hemisphere. The distribution of asymmetry scores for this trait, averaged across three ratings, is shown in Figure 2 and the tabulated summary by sex and range is shown in Table 1. Two brain images could not be scored due to insufficient sulcal features and one individual was removed from the analysis because of a pathologically small right prefrontal lobe (see below). Scores ranged from −2 indicating strong asymmetry with right sulcus anterior to the left to +2 indicating strong asymmetry with left sulcus anterior to the right. About one third (38%) of the images showed no difference in the relative position of the cingulate sulcus and were consistently scored as zero across all three ratings. An additional 20 images (5.6%) were ambiguous with respect to asymmetry, receiving two 0 scores and a single score of either −1 or +1. Just over half of the animals (182 animals, 51.4%) showed asymmetry with the left sulcus anterior to the right (mean score >+0.33) and only a small proportion (17 animals, 4.8%) showed asymmetry in the opposite direction (mean score <−0.33). We examined the pedigree structure and relationships between the 17 individuals who showed atypical asymmetry, but did not identify any evidence to support the possibility that this trait was influenced by a major locus. We therefore defined the cingulate sulcus asymmetry phenotype as a dichotomous trait for subsequent heritability estimation analysis using a liability threshold model by setting a threshold at a score of +0.33 (dashed line in Figure 2). A higher proportion of females were scored as asymmetric for this trait, which is reflected in the higher mean score for females and permutation testing showed that the difference was statistically significant (empirical p-value of 4.7×10^−3^). We tested whether the difference observed between males and females was simply related to the difference in brain size, using a linear model to regress total brain volume on the cingulate sulcus trait, but did not find a significant effect.

**Table 1:** Average scores for cingulate sulcus asymmetry by sex and range.

	n	mean score	−2≤n<−0.33	−0.33≤n≤+0.33	+0.33≤n≤+2
males	101	+0.44	9 (8.9%)	52 (51.5%)	40 (39.6%)
females	253	+0.73	8 (3.2%)	103 (40.7%)	142 (56.1%)
total	354	+0.65	17 (4.8%)	155 (43.7%)	182 (51.4%)

#### 1.2 Hemisphere Volume and Cerebral Widths

The distribution of the AQ for hemispheric volume is shown in the top graph of the right panel in Figure 3. There was no effect of sex or age on the AQ. One individual was clearly an outlier with an asymmetry quotient over six standard deviations from the mean and almost 2.5 standard deviations from the next highest individual. Examination of the brain image for this individual showed a pathologically small right prefrontal lobe. Detailed records from the research colony did not indicate any obvious medical or behavioral differences in this individual. Data for this animal were removed from subsequent statistical and variance component analysis. Permutation testing showed that the sample distribution of the asymmetry quotient did not differ significantly from the null distribution indicating that there was no pedigree-level asymmetry with respect to hemispheric volume.

The AQ distributions for occipital and prefrontal widths are shown in the right panel of Figure 3 and summarized in Table 2. There was no effect of age or sex on the AQ for either region. The distribution of AQ scores for the prefrontal region was more variable as indicated by a coefficient of variation of 17.12 compared to a coefficient of variation for the occipital region of 2.25. The mean AQ for the occipital region was positive and permutation testing showed it was significantly different from that expected under the null hypothesis, indicating a pedigree-level asymmetry bias toward wider right occipital lobes in the VRC sample. The mean AQ for the prefrontal region was negative suggesting a leftward bias, but permutation testing did not reach significance for the hypothesis that the mean differed from the null hypothesis. The raw scores for the left and right hemispheres widths were strongly correlated within the prefrontal and occipital regions, but only weakly correlated between the two regions (Table 3). The AQs for the prefrontal and occipital regions were not correlated (Table 4).

**Table 2:** Summary table for asymmetry phenotypes. Permutation testing was used to determine p-values. sd, standard deviation.

Phenotype	mean	sd	p-value
Hemisphere volume AQ	9.07E-5	1.56E-2	0.91
Prefrontal width AQ	−4.83E-3	8.27E-2	0.27
Occipital width AQ	1.67E-2	3.75E-2	<1.0E-05
Transverse skew	−3.54E-3	4.57E-3	<1.0E-05
Coronal skew	8.26E-4	5.24E-3	2.83E-3

**Table 3:** Correlation coefficients of cerebral widths for each pair of regions.

	Left Prefrontal	Right Prefrontal	Left Occipital	Right Occipital
Left Prefrontal	—	0.50	0.18	0.18
Right Prefrontal	<1.0E-05	—	0.16	0.15
Left Occipital	4.6E-4	3.0E-3	—	0.75
Right Occipital	5.3E-4	4.7E-3	<1.0E-05	—

The Pearson's correlation is shown in the upper right triangle of the matrix and the p-value determined from permutation testing is shown in the lower left triangle of the matrix.

**Table 4:** Phenotype correlations among the asymmetry measures.

	CingulateSulcus	HemisphereVol. AQ	PrefrontalWidth AQ	OccipitalWidth AQ	TransverseSkew	CoronalSkew
Cingulate Sulcus	—	0.01	0.00	−0.07	−0.25	−0.14
Hemisphere Vol. AQ	0.79	—	−0.02	0.10	−0.22	−0.03
Prefrontal Width AQ	0.95	0.62	—	−0.01	0.08	0.19
Occipital Width AQ	0.20	0.06	0.79	—	−0.01	0.02
Transverse Skew	<1.0E-05	4.0E-5	0.12	0.91	—	0.25
Coronal Skew	7.5E-3	0.63	2.2E-4	0.74	2.0E-5	—

The correlation estimate is shown in the upper right triangle of the matrix and the p-value based on permutation testing is shown in the lower left triangle of the matrix.

#### 1.3 Skew

The amount of skew in the transverse and coronal planes required to optimally register each brain image to a symmetric atlas template is a measure of left-right asymmetry. The distributions of the transverse and coronal skew are shown in Panel C of Figure 4. The sample mean for the transverse skew was negative and permutation testing indicated that it was significantly different from that expected under the null distribution (Table 2), demonstrating a pedigree-level shift of left hemisphere volume anteriorly relative to the right and a posterior shift of volume in the right hemisphere compared to the left. (Figure 4). The sample mean for the coronal skew was positive and significantly different from that expected under the null distribution indicating a dorsal shift of left hemisphere volume compared to the right and a ventral shift of volume in the right hemisphere compared to the left. Age, sex and total cerebral volume did not have any significant effect on either measurement. The orthogonal skews were significantly correlated with each other (r = 0.25, p-value = 2.0×10^−5^).

### 2 Correlation among Asymmetry Measures

The correlation coefficients and p-values based on permutation distributions for the 15 pairs of asymmetry measures are shown in Table 4. There were four significant correlation coefficient estimates including the previously noted relationship between coronal and transverse skew. Additionally, transverse skew showed moderate correlations with the cingulate sulcus trait (r = −0.25) and the asymmetry quotient for hemisphere volume (r = −0.22). The measure of coronal skew was correlated with the AQ for prefrontal width (r = −0.19) and also showed a trend toward a low correlation with the cingulate sulcus trait, although this result did not meet standard thresholds for statistical significance after correcting for multiple tests.

### 3 Variance Component Analysis of Genetic and Environmental Influences

Prior to estimating the genetic contributions to the AQs, we implemented variance component analysis to estimate the proportion of variance due to genetic (heritability estimate) and environmental influences on cerebral volume and width measurements from the left and right hemispheres (upper portion of Table 5). The heritability estimate for the volume of each hemisphere was greater than 0.90. Likewise the heritability for occipital width was moderately high with similar estimates for the right (h^2^ = 0.54, se = 0.10) and left hemispheres (h^2^ = 0.52, se = 0.11). In contrast, the heritability estimates for the left and right prefrontal widths differed. Whereas the left prefrontal width was significantly heritable with an estimate of 0.40 (se = 0.11), the estimate for the right prefrontal width was lower (h^2^ = 0.17, se = 0.10) and did not reach our threshold for significance. Bivariate analysis was implemented to estimate the proportion of covariance for each pair of cerebral widths that was due to common genetic causes (i.e. genetic correlation) and environmental causes (i.e. environmental correlation). The results are listed in Table 6 and show that most of the genetic sources that contribute to each hemisphere are shared (common genetic sources), but most of the environmental sources that contribute to variance are not shared. The proportion of covariance due to environmental sources between the two sides of the occipital region was significantly higher than the estimated proportion between the right and left prefrontal regions (0.42 vs. 0.21).

**Table 5:** Heritability estimates. se, standard error of the estimate.

phenotype	h^2^	se	p-value
VOLUME & WIDTH PHENOTYPES			
Left Hemisphere Volume	0.94	0.06	1.3E-22
Right Hemisphere Volume	0.97	0.06	8.8E-23
Left Prefrontal Width	0.40	0.11	4.0E-6
Right Prefrontal Width	0.17	0.10	0.026
Left Occipital Width	0.52	0.11	2.0E-7
Right Occipital Width	0.54	0.10	2.1E-8
ASYMMETRY PHENOTYPES			
Cingulate Sulcus	0.32	0.23	0.012
Hemisphere Volume AQ	0.18	0.10	0.021
Prefrontal Width AQ	0.02	0.07	0.41
Occipital Width AQ	0.00	—	0.50
Transverse Skew	0.29	0.10	1.7E-4
Coronal Skew	0.10	0.09	0.08

**Table 6:** Genetic (**ρ_G_**) and environmental (**ρ_E_**) correlations for cerebral width phenotypes.

ρ_E_\^ρ^_G_	Left Prefrontal	Right Prefrontal	Left Occipital	Right Occipital
Left Prefrontal	—	0.97 (0.18)	0.55 (0.16)	0.46 (0.16)
Right Prefrontal	0.21 (0.10)	—	0.55 (0.25)	0.53 (0.25)
Left Occipital	−0.16 (0.15)	−0.02 (0.12)	—	0.99 (0.04)
Right Occipital	−0.09 (0.14)	−0.03 (0.12)	0.42 (0.11)	—

The estimate of the proportion of common genetic sources contributing to the phenotypic covariance of each pair of cerebral width is shown in the upper triangle of the table and the proportion of common environmental sources contributing to the covariance between traits is shown in the lower triangle of the table. The standard error of the estimate is in parentheses.

Heritability estimates for the AQs are shown in the lower portion of Table 5. The only asymmetry measure that met a Bonferroni corrected significance threshold of 8.33×10^−3^ was transverse skew (Table 5). Two other asymmetry measures showed trends for significant heritability; the relative position of the ascending ramus of the cingulate sulcus and the hemisphere volume asymmetry quotient.

Because the majority of variance for each asymmetry phenotype was due to environmental sources of variance, we examined the effect of a range of maternal and other environmental factors on each phenotype. We assessed the possible influence of gestational environment by estimating the effect of shared environment among offspring from the same mother, but did not identify any significant estimates of shared environmental effect for any of the phenotypes analyzed in this study. Using linear models, we tested the effect of maternal weight, maternal age, month of birth, and measures of maternal-infant interaction on each of the asymmetry phenotypes, but no factor achieved significance at a threshold accounting for a Bonferroni correction.

## Discussion

Most of the asymmetry literature has focused on the characterization of lateralized behavior. A much smaller number of investigations have been dedicated to the characterization of anatomic asymmetry and an even smaller fraction has attempted to study inter-individual variance for asymmetry traits. Non-human primate model systems are a potentially powerful resource for these studies, but to date, outside of great apes, have provided equivocal evidence regarding the extent and direction of anatomic asymmetry. In the current study, we leverage the power of a large sample of high-resolution brain MRIs acquired from an extended pedigree of vervet monkeys to make two general contributions to the study of anatomic asymmetry in primates. First, we provide significant evidence for pedigree-level directional bias in four of six measures of anatomic asymmetry in the VRC. Second, we estimated the environmental and genetic components of inter-individual variance for each phenotype, and show that about 30% of variance for anatomic asymmetry is due to genetic causes.

Of the six symmetry phenotypes, only hemisphere volume AQ and prefrontal width AQ did not demonstrate pedigree-level asymmetry. The fact that hemisphere volume, unlike most of the other measures, did not show any pedigree-level bias suggests that anatomic asymmetry arises from a differential distribution of cerebral volume within the two hemispheres. Taken together, the skews in the coronal and transverse plane indicate that the cerebral volume is distributed asymmetrically along both the anterior-posterior (AP) and dorsal-ventral (DV) axis. Compared to the left hemisphere, cerebral volume in the right hemisphere is shifted posteriorly along the AP axis and ventrally along the DV axis, whereas left hemisphere volume is shifted anteriorly and dorsally compared to the right. The transverse and coronal skews are moderately correlated with each other (r = 0.25) indicating that the differential distribution of volume along the two orthogonal axes tends to occur together, but is not tightly coupled. Besides the skew measures, the other two asymmetry traits that showed pedigree-level bias support the general pattern of asymmetry described above. The posterior shift of volume in the right hemisphere is reflected in the pedigree-level tendency toward a wider right occipital lobe and in the finding that the ascending ramus of the right cingulate sulcus was shifted posteriorly on the AP axis in a majority (91.5%) of the animals with asymmetric cingulate sulci. The methods implemented in this study are relatively general, and do not provide much information regarding asymmetry that may be localized to specific regions. Methods have been developed to measure the asymmetry quotient from gray-matter maps at a voxel-sized scale along the AP and DV axis (e.g. Barrick et al. [24]). These methods pose a multiple testing problem for genetic analysis, but allow for the possibility of ‘fine mapping’ asymmetry to specific anatomic structures with known behavioral correlates that may inform hypothesis about the functional significance of the asymmetry.

The pattern of asymmetry that we identified contrasts with the common pattern of anatomic asymmetry observed in humans, who have a population bias with volume shifted anteriorly in the right hemisphere and posteriorly in the left hemisphere. Most of the previous research on endocranial shapes has shown that chimpanzees and gorillas have a biased asymmetry similar to humans. However, a recent study using innovative three-dimensional methods to quantify asymmetry from endocasts found a majority of gorilla skulls in their sample demonstrated a rightward biased asymmetry in the occipital regions [5]. This pattern is in the same direction as the current findings in vervets and support Balzeau et al.'s hypothesis that methodologies deigned to precisely measure asymmetry in three-dimensional space can identify subtle symmetries that previous, largely qualitative methods, were unable to detect. More generally, these findings underscore the importance of characterizing anatomic brain asymmetry without *a priori* expectation of directional bias. Emerging evidence in comparative behavior suggest the possibility of a general trend across a broad range of species including fish, frogs, birds and mammals for right-hemisphere specialization for approach behaviors and left-hemisphere specialization for avoidance behaviors [25]–[27]. The growing evidence for similarities in patterns of lateralized behavior has motivated a search for common patterns of anatomic asymmetry among species. However, an agnostic approach is preferable. A consistent trend in the directional bias of lateralized behavior across a wide range of species does not obligate a similar trend in the directional bias of brain asymmetry. Different species may employ alternate mechanisms to achieve lateralized behaviors that may manifest in different patterns of anatomic asymmetry, and may even account for the mixed findings for the directional bias of brain asymmetry among species of macaques. However, as noted in the Introduction, previous studies of primate brain been characterized by substantial heterogeneity in sample size and protocols for measurement, and considerable additional investigation will be required to accurately compare patterns of asymmetry across species.

The functional significance of anatomic asymmetry in non-human primates is an important overarching question for the field. It is difficult to speculate about the functional significance of our findings. Even the cingulate sulcus trait, which is the most anatomically localized measure we employed, may represent a sensitive indicator of global volumetric shifts due to its central location at the midline. An additional limitation on functional hypotheses is the uncertainty regarding cytoarchitectonic similarities between vervets and macaques, for which detailed functional maps are available (see Woods et al. [28] for discussion of comparative brain anatomy in Old World monkeys). In macaques, the cingulate sulcus is bordered by regions PE and PEci (rostral, dorsal), PEc (caudal, dorsal), and PGm (caudal, ventral). These regions integrate visual and limb movement information with motor regions of the frontal cortex suggesting they generate a representation of one's own body and integrate whole body interactions with the visual environment including hand-eye coordination [29]–[32]. The hypothesis that cingulate sulcus asymmetry is correlated to lateralized behavior could be tested in the VRC using a hand-preference task.

Among the asymmetry measures, only transverse skew showed statistically significant evidence of heritability. The estimate of heritability for the dichotomous cingulate sulcus measure was similar in magnitude, but with a higher standard error compared to the estimate for transverse skew. In part this finding may be due to the better power of a continuous trait using the variance-component analysis method that we employed. It may also reflect an advantage of atlas-based methods, which use voxel data from the entire cerebral volume to linearly deform each image to match a symmetric atlas template and provide measures of global features that complement regional or localized measures like volume and width. Although the heritability estimate for transverse skew was significant, it was much lower than that observed for other neuroimaging phenotypes in the VRC [20]. Although about 70% of the variance for this measure can be attributed to non-genetic sources, we have not yet identified the environmental factors that could account for this variance.

Although the genetic isolation and large complex pedigree structure of this sample adds power for genetic analysis, it must be emphasized that this dataset does not represent a population sample of independent individuals, and therefore the results cannot be generalized to other populations. The current work provides a rigorous investigation of anatomic asymmetry in a non-human primate genetic model system and demonstrates that it is possible for the primate brain to develop an asymmetry pattern quite different from that typically observed in humans. Although, the heritability estimates observed in this study cannot be extrapolated to study samples outside of the VRC pedigree, they provide the most conclusive evidence to date that genetic factors can contribute to inter-individual variance in anatomic asymmetry. Combined with growing evidence of anatomic asymmetry in other Old and New World monkeys [11]–[14], [33], our findings strongly challenge the hypothesis that anatomic brain asymmetry is uniquely human. As an alternative, it suggests that anatomic asymmetry is a quantitative trait that lies on a continuum in primates, and its characterization across species may provide important insights into primate brain evolution.
